# High-pitch sounds small for domestic dogs: abstract crossmodal correspondences between auditory pitch and visual size

**DOI:** 10.1098/rsos.211647

**Published:** 2022-02-09

**Authors:** A. T. Korzeniowska, J. Simner, H. Root-Gutteridge, D. Reby

**Affiliations:** ^1^ School of Psychology, University of Sussex, Brighton, BN1 9RH, UK; ^2^ School of Life Sciences, University of Lincoln, Lincoln, Lincolnshire, UK; ^3^ Universite Jean Monnet Saint-Etienne, Saint-Etienne, Rhône-Alpes, France

**Keywords:** dog, crossmodal, pitch, size, correspondences

## Abstract

Humans possess intuitive associations linking certain non-redundant features of stimuli—e.g. high-pitched sounds with small object size (or similarly, *low*-pitched sounds with *large* object size). This phenomenon, known as crossmodal correspondence, has been identified in humans across multiple different senses. There is some evidence that non-human animals also form crossmodal correspondences, but the known examples are mostly limited to the associations between the pitch of vocalizations and the size of callers. To investigate whether domestic dogs, like humans, show abstract pitch-size association, we first trained dogs to approach and touch an object after hearing a sound emanating from it. Subsequently, we repeated the task but presented dogs with *two* objects differing in size, only one of which was playing a sound. The sound was either high or low pitched, thereby creating trials that were either congruent (high pitch from small object; low pitch from large objects) or incongruent (the reverse). We found that dogs reacted faster on congruent versus incongruent trials. Moreover, their accuracy was at chance on incongruent trials, but significantly above chance for congruent trials. Our results suggest that non-human animals show abstract pitch sound correspondences, indicating these correspondences may not be uniquely human but rather a sensory processing feature shared by other species.

## 1. Introduction

Humans intuitively pair high-pitched sounds with small objects and low-pitched sounds with large objects. This phenomenon is referred to as the *pitch-size crossmodal correspondence* and is just one of a number of low-level crossmodal correspondences discovered in humans to date. For example, humans also have implicit preferences linking gustation and audition (e.g. sour tastes map to sounds with higher spectral frequency than sweet tastes; [1]), audition and vision (e.g. lighter colours are preferentially paired with higher pitch sounds; [2]), and vision and haptics (e.g. softness maps to colours that are higher in chroma/luminance than hardness; [3]; for review see [4]). One of the most well-documented correspondences is the pitch-size correspondence noted above (pairing higher pitch with smaller objects). First mentioned in 1883 by Stumpf (cited in [5]), the developmental mechanism behind this is still a matter of debate—but it may be the result of early post-natal learning, as evidenced by the fact that this particular correspondence can be found in 6- and 36-month-old babies, but not earlier at 4 months of age ([6,7]; but see [8]). Cumulatively, these data suggest that some experience of the world outside the womb, or perhaps maturation of the cortex at that point, is necessary for the pitch-size correspondence to arise. But whether developed pre-natally or emergent through learning after birth (or indeed some combination of both), a relationship between pitch and size has been known for some time. This is illustrated in the early work of Von Bekesy [9], who demonstrated as early as the 1950s that size perception is affected by auditory stimulation, i.e. increasing the auditory frequency of a sound stimulus was perceived as the stimulus decreasing in size.

If crossmodal correspondences are acquired via learning after birth, how might humans learn them? World statistics are certainly a possible source, i.e. the likelihood of certain stimulus pairings in the environment. For example, one basis for this association might be the physical properties of inanimate objects, such as the frequencies at which they resonate when struck (e.g. small versus large bell, see [10]). Similarly, large animals such as elephants tend to produce low-pitched vocalizations due to larger vocal folds which oscillate at lower frequencies, while smaller animals, such as mice, squeak at high frequencies (for review see [11]). The exposure to such regularities in the environment could result in a learnt association between size and pitch. What's more, being able to predict the size of a sound-producing stimulus would have a clear adaptive value to individuals trying to make sense of their environment [4]. This suggests in turn that being able to form pitch-size crossmodal correspondences could be a useful and adaptive quality for *all* animals, not just humans. Hence, we here explore whether there is evidence for the pitch-size correspondence in non-human animals, testing their occurrence in domestic dogs.

There is already some evidence that non-human animals are capable of crossmodal correspondences, at least in certain restricted circumstances (for review see [12]). First and foremost, animals use conspecific vocalizations to assess the size of a vocalizing individual, and this has been found in classes ranging from fish (e.g. [13]) to anurans (e.g. [14]) through to mammals (e.g. [15]). There is also some evidence that chimpanzees may use higher pitched vocalizations to refer to smaller trees (and low pitch for large trees [16]). But whether (non-human) animals form true abstract correspondences is a very different question (i.e. audio-visual correspondences *abstracted away from animal calls*, e.g. matching low-pitched abiotic sounds with large objects). Evidence from chimpanzees suggests they may in certain circumstances: in a match-to-sample experiment, chimpanzees were more accurate matching two luminant (i.e. light-coloured) squares in the presence of an irrelevant high-pitched sound (and better at matching dark squares alongside low-pitched sound) [17]. This shows evidence of a true correspondence, at least for pitch-luminance. Domestic chicks also appear sensitive to luminance, showing a luminance-space correspondence as early as at 3 days old [18], i.e. they preferentially paired the left side of space with low (dark) luminance and the right-hand space with high (light) luminance. This again suggests crossmodal correspondences involving luminance might be a feature of perceptual systems in some non-human (primate and avian) species. However, there are no similar data on the correspondence of pitch-to-size.

Here, in order to further investigate the universality of correspondences, we tested the pitch-size correspondence in domestic dogs, a species of large terrestrial mammals with a long history of domestication (e.g. [19]). As with many other animals (see discussion above), dogs can use sound to assess the size of vocalizing conspecifics [20,21]. However, studies have focussed on formant dispersion as the relevant cue (corresponding to vocal tract resonances and thus vocal tract length) rather than pitch (corresponding to fundamental frequency and thus vocal fold length) which is less size dependent between individuals of the same age or size classes [20]). This is in line with other mammalian species [15], despite the fact that dogs—in particular—could theoretically use pitch as an informative cue to size, given the vast size differences across breeds which could lead to size-related variation in pitch (F0) [22]. Another reason why dogs are a good candidate for our investigation is the fact that recently we found that they show evidence for a crossmodal correspondence between acoustic pitch and elevation (spatial height). In our earlier study, they preferentially attended to pitch-elevation pairings that were *congruent*, as opposed to *incongruent* [23]—where ‘congruency’ is defined as matching human intuition (i.e. congruent = high-pitched sounds were associated with high spatial elevation, and low-pitched sounds with objects near the ground; incongruent = the reverse). Our findings of a congruency effect were the first evidence for an abstract crossmodal association in a non-primate and the first evidence of pitch-elevation correspondences in any non-human animal.

In the current study, we investigated whether domestic dogs are capable of forming the pitch-size crossmodal correspondence. We designed a paradigm in which we trained dogs to approach one of two presented objects (large or small), always choosing the object that was emitting a sound (high or low pitched). We predicted that dogs would tend to choose the correct object (i.e. the one emitting a sound) more often on congruent trials (where the sound frequency would match the size of the object that the sound was coming from). Finally, as well as accuracy differences, we also predicted that dogs during congruent trials might react faster; i.e. show shorter latencies to move and take less time to approach the object.

## 2. Methods

### 2.1. Participants

Fifty domestic dogs of unrestricted breeds aged between 6 and 132 months (*M* = 56.1, s.d. = 36.6) who had previously received some basic trainings (e.g. basic commands such as ‘sit’ and ‘stay’) were recruited through social media advertisements and word of mouth. We removed dogs who failed to complete training (*n* = 8), or who failed to produce any useable testing trials (*n* = 12; e.g. they were too distracted to perform the task). A further 111 trials from the remaining dogs were also removed (e.g. due to background noise, or owner interference; see electronic supplementary material for full exclusion criteria). This left 133 trials from 30 dogs.

### 2.2. Materials

**Figure 1.**
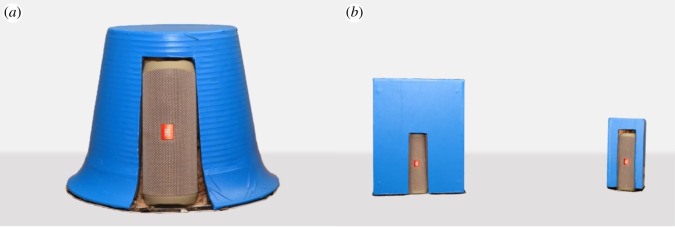
Visual training and testing stimuli: (*a*) Back view of the frustum used in the training phase of the experiment. (*b*) An example of the pair of shapes (here, cuboids) used in the testing phase of the experiment (*a*) and *b*) are not to the same scale). Dogs saw the front side of the objects during testing (i.e. the speaker was not visible to them).

**Figure 2.**
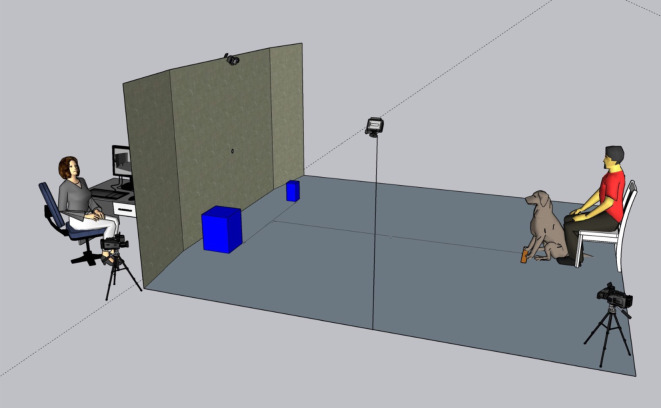
The set-up of the laboratory in the testing phase. One of the two blue objects emits a sound (here we show the example of two cuboids), and the dog is trained to touch this sound-emitting object with paw or nose. For a full description of the elements in this scene, please see main text. During the training phrase, the set-up was identical except that there was a single mid-sized object (a blue frustum; figure 1).

The training stimulus consisted of a plastic-coated three-dimensional frustum-shaped object, painted blue (figure 1*a*) to increase visual salience for the dogs [24]. The frustum had a base diameter of 29 cm and a volume of approximately 9000 cm^3^. A JBL Flip 4 Bluetooth speaker was inserted via a small hole in the side of the object (figure 1*a*) and the remaining cavity filled with insulating rockwool (Knauf Earthwool) to reduce any resonances from the speaker. The sound stimulus for training was a 2 s-long 430 Hz (9.33 erb) pure tone created in PRAAT [25]. The sound was played at 86 dB (approximately 65 dB measured at the dog's position) which was controlled from a Motorola Razor phone connected to the speaker via Bluetooth. The dogs' behaviour was recorded using three cameras: a Go Pro Hero 7 camera positioned on the right side of the dog, a GoPro Max camera positioned to the front of the dog, and a Sony Handycam camera positioned to the right (or left, depending on the position of the experimenter) and behind the dog. Additionally, there was a Sony Handycam camera, positioned in front of the dog, to the right or left of a partition screen which was hiding the experimenter (figure 2). This was connected to an Acer LED 21.5″ Monitor (KA220HQ) where the experimenter could watch the behaviour of the dog while out of sight.

The testing stimuli were identical to training stimuli in all ways other than the following: there were now *two* objects per trial: one big and one small (approximately 23 000 cm^3^ and 2300 cm^3^, respectively). To ensure that our findings would hold across multiple shapes, we created three sets of objects: a pair of cylinders, a pair of cones and a pair of cuboids (for this latter figure 1*b*)—ensuring each dog saw one set only (e.g. a big and small cuboid). There were now also two sound stimuli, one low pitch (150 Hz, 4.26 erb) and one high pitch (900 Hz, 14.4 erb). All other aspects of stimuli and recordings were as in the training phase.

Remaining materials were a partition screen, button clicker, reward tube and rewards. The experimenter trained the dogs to touch the object using a button clicker and commercially available dog treats (see below). Treats were delivered via the plastic reward tube attached to the partition screen, whose position and orientation is described below.

### 2.3. Procedure

The laboratory set-up is illustrated in figure 2.

### 2.4. Training

Dogs were brought into the testing room at the University of Sussex by their owners and given approximately 5 min to acclimatize, while owners signed the consent form and completed a questionnaire about their dogs (stating their breed, age, sex, neuter status and weight). In order to avoid any potential bias resulting from their expectations, the owners were not informed about the purpose of the study or the hypothesis until testing was completed, at which point they were debriefed. Then, the owners were asked to sit in a chair facing a partition screen (figure 2), which had a reward tube attached to it (shown as the small hole in the centre of the partition in figure 2). Each dog was put into a ‘sit’ position directly in front of the owner to prevent the owner from accidentally cueing the dog, and owners were asked not to interact with their dogs during the experiment. The dog was positioned directly in front of the object at the start of training. The training object, a mid-sized blue frustum, was positioned between the dog and the screen, 90 cm away from the screen.

When training began, the dog underwent a shaping phase with the aid of a clicker to train the dogs to target the training object with their nose or paw. This technique involves waiting for the desired behaviour and quickly marking and rewarding when it occurs. This method was chosen to avoid additional prompts or social cues that would later have to be faded [26], which could prolong the training process. The primary reinforcer (food treat) was delivered through a reward tube attached to the screen positioned behind the object (facing the dog). After a correct response was established (i.e. dog consistently targeting the object and touching with nose or paw), a sound playing from the speaker within the object was now introduced. If the dog moved too soon (before the sound was played), the experimenter prevented the dog from touching the object, by blocking it with her hand or lifting the object up. After each repetition of the behaviour, the dog was returned to the ‘sit’ position in front of the owner and another trial was initiated.

As the training progressed, the owner and the dog were slowly moved backwards to increase the distance the dog had to travel to touch the object, and the experimenter started to gradually retreat behind the room-dividing screen which was necessary in order to avoid influencing the dogs' behaviour during testing. Training continued until the owner's chair was in position by the wall opposite the screen (350 cm away from the screen), *and* the dog was able to sit and wait in an appropriate position for the next sound to be played by the experimenter (now hidden out of view behind the screen). The appropriate position for the dog was to be seated in front of the owner but behind an orange line drawn on the floor. A final six trials (object to the left of the dog ×2, object to the right of the dog ×2, object in the middle ×2) had to be completed with 100% accuracy before training ended and the experiment moved on to the testing phase. If this failed, another block of six training trials was completed.

### 2.5. Testing

Testing took place immediately after successful training was complete, and the dog and owner remained in the position they had reached at the end of training. All elements of testing were identical to training except now two objects were used, again placed in front of the screen, one to the left and one to the right, equidistant from the reward delivery tube, 90 cm in front of the screen, 124 cm away from each other and 150 cm away from the dog (figure 2). The shape of the objects was randomly assigned to each dog (i.e. a pair of cylinders or cones or cuboids). Only one of the objects played a sound on each trial. Dogs were presented with eight trials in total, crossing the three variables shown in table 1. This crossing effectively means every trial presented two objects (big and small): one of which was playing a sound (high or low pitched), with objects switching on the left or right of the space (e.g. Trial 1 big-on-left; Trial 2 big-on-right). The presentation of two objects and two sounds was necessary because the pitch-size correspondence appears to be relative in humans, rather than absolute [27]. In other words, we hypothesized that dogs must see two objects to know that one is ‘big’ and must hear two sounds to know that one is ‘high’.^1^

**Table 1.** Variables crossed to produce eight trials per dog. Sounds are emanating from the objects, but only one object makes a sound in each trial. The positioning variable describes the positioning of each object, either side of the reward tube (figure 1).

sound frequency	object size	object position
high pitch	large	left
		right
	small	left
		right
low pitch	large	left
		right
	small	left
		right

As with training, each trial began with the experimenter positioning the stimuli (i.e. moving the small/big objects left/right, according to the trial type) and setting the dog up in a sitting position in front of (and facing away from) its owner. The experimenter then retreated behind the screen, from where she could manipulate the sound media. She then activated the sound, which was the signal for the dog to approach and touch the sound-making object. When the dog touched the object, the experimenter marked the behaviour with a click from the clicker and deposited a small treat via the tube as a reward for the dog. The dog was then repositioned by the experimenter in preparation for the next trial. If the dog did not respond at all within 45 s from start of the sound stimulus, the sound was repeated. If the dog continued to not respond, the trial was recorded as ‘no response’, and the next trial began. If the dog moved out of position, but made no choice (e.g. went to investigate an area of the testing room where there was no object), this was also recorded as ‘no response’. If the dog showed signs of stress such as excessive panting, pacing, comfort-seeking from owner, excessive whimpering, or avoidance of the experimenter, the experiment was terminated.

### 2.6. Design

Of each dog's eight trials, four were congruent (small object playing high-pitch sound; or big object playing low-pitch sound; once with small object on the left and once with small object on the right). The remaining four trials were incongruent (i.e. small/low on left and right, and big/high on left and right). The combination of trials was pseudo-randomly assigned to each dog, to avoid any ordering effect.

### 2.7. Video coding

Video recordings of dog behaviour were edited in iMovie (Apple Inc.) to replace the sound stimulus for each trial with a ringing tone to allow the behaviour of the dogs to be coded blind. Subsequently, the recordings were analysed using SportsCode Gamebreaker version 10 by ATK, and 25% of trials by HRG.

## 3. Results

Our independent variable was whether the trial was congruent or incongruent. For this, we examined three dependent variables: (i) the dogs' accuracy (i.e. ability to choose the sound-making object), (ii) speed of approach to objects (measured as the time in seconds between their front leg crossing the orange start-line in front of them, and the moment they touched one of the objects) and (iii) their latency to move (measured as the time from the start of the sound stimulus to when the dog moved off the spot). Linear mixed models (LMM) with Dog ID as a random effect were run for the continuous outcomes and general estimating equations (GEE) were run for the binary outcomes using SPSS v. 26 (IBM Corp.), with alpha level set at 0.05 for both. For comparison, 25% of trials (*N* = 33) were double coded by HRG and inter-rater agreement calculated using Cronbach's alpha was high for both latency to move (1.0) and speed of approach (0.93). Additionally, there was a 100% agreement on the accuracy measure.

### 3.1. Accuracy

**Figure 3.**
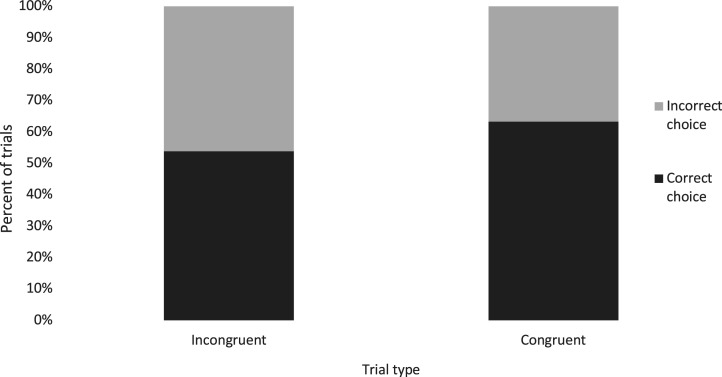
Percentage of trials where dogs chose the correct object, split by trial type. Dogs were better than chance at choosing the correct object on congruent trials but at chance on incongruent trials.

The difference in accuracy (i.e. the ability to choose the sound-producing object) between congruent and incongruent trials was not statistically significant (binary logistic GEE, *B* = 0.386, s.e.(B) = 0.33, OR = 1.47, *p* = 0.24). However, when compared to chance responding, dogs chose a correct object significantly above chance on congruent trials (binary logistic GEE, *B* = −0.572, s.e.(B) = 0.21, OR = 0.564, *p* = 0.006), while their accuracy was at chance on incongruent trials (binary logistic GEE, *B* = −0.161, s.e.(B) = 0.26, OR = 0.851, *p* = 0.53). This suggests that size-sound matching affected the dogs' ability to locate the sound figure 3.

### 3.2. Speed of approach

There was no effect of the trial type (congruent or incongruent) on the speed of approach to the object (LMM, *F*_1,109.300_ < 0.0001, *p* = 0.983). Dogs approached the object of choice with the same speed regardless of whether the trial was congruent or incongruent (M(congruent) = 3.5s, s.e. = 0.308; M(incongruent) = 3.5s, s.e. = 0.310). We found, however, that dogs approached the correct object (the one making a sound) faster (*M* = 3.23s, s.e. = 0.30) than they approached the incorrect object (*M* = 3.90s, s.e. = 0.33), and this was significant (LMM, *F*_1,118.42_ = 4.27, *p* = 0.041). This demonstrates that our training techniques (teaching dogs to target sound-producing objects) had worked successfully.

### 3.3. Latency to move

**Figure 4.**
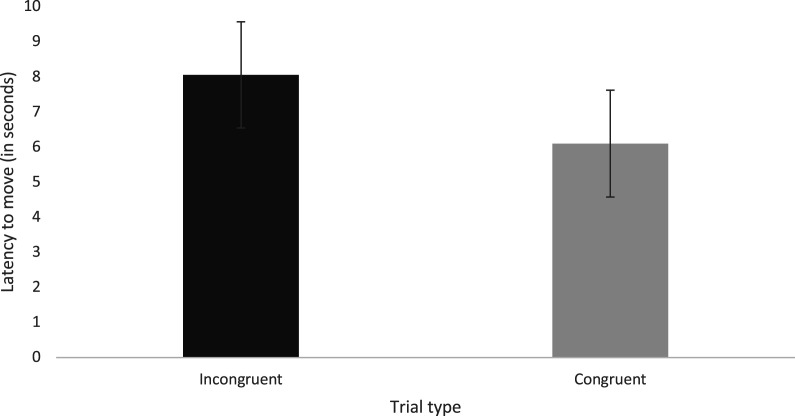
Dogs were significantly quicker to react to the sound stimulus on the congruent trials compared to incongruent trials (error bars represent ± s.e.m.).

An LMM revealed that the type of trial (congruent versus incongruent) significantly affected the dogs’ latency-to-move i.e. the time from the start of the auditory stimulus to the time they started responding (e.g. by lifting their body up from the sitting position) (*F*_1,90.708_ = 4.723, *p* = 0.032). Dogs were significantly slower to react on incongruent trials (M(incongruent) = 8.06s, s.e. = 1.51) compared to congruent trials (M(congruent) = 6.01s, s.e. = 1.52). This suggests that the congruency of the trial affected how quickly the dogs made the decision to respond to the auditory stimulus figure 4.

## 4. Discussion

Here, we investigated whether dogs spontaneously associate pitch and size, using artificial audio-visual stimuli. We found that dogs were indeed sensitive to this congruency in audio-visual processing. When responding to geometric objects emitting sounds, dogs were faster to initiate movement when pitch was congruent to the size of the object (high pitch from small objects; low pitch from large objects). Also, when the pitch was congruent, dogs were significantly better than chance at detecting the sound-emitting object, but they were no better than chance in the incongruent condition. Finally, there was no difference in the speed to approach (other than dogs being faster when they were correct). In summary, however, two of three measures suggested that domestic dogs are sensitive to the same crossmodal pitch-size correspondences as found in humans.

Since our stimuli were artificially generated pure tones and arbitrary shapes, rather than conspecific vocalizations (as in [20,21]), our findings show that dogs have an abstract concept of this correspondence independent of vocalizations, suggesting a more generalized perceptual phenomenon. Our finding has ecological validity in that this abstract knowledge could influence animals' behaviours in real-world settings. Although acoustic allometry studies have tended to focus on formant frequency as the relevant cue when determining the size of vocalizing conspecifics (for both for dogs [20] and other mammals (e.g. pigs [28]), pitch itself (F0) is also an informative cue, especially in the widely varying morphology of dogs ([22]; see Introduction). Indeed, since formant dispersion and F0 *both* vary in small to large dogs’ vocalizations ([21,29], Farago and colleagues have suggested that *both* could act as a cue to size in dogs ([21]; see also [11] for other mammals). We must also consider the relevant ecological fact that dogs are perhaps the closest non-human domestic companion to humans themselves. It may therefore be highly relevant that dogs show a preference to match high-pitched sounds with small size because this mirrors the intuitions of humans themselves. Adult humans respond faster to congruent size-sound parings [30,31], and even young children match pitch and size according to the predictions of the pitch-size correspondence [6]. Hence, a similar correspondence in dogs (as shown in own study) could potentially aid in cross-species communication, and we are now testing this theory directly by examining dog-directed human speech. A final ecological application of our results is the non-trivial suggestion that pitch-size correspondences could aid dogs in determining the age (and in adults the sex) of vocalizing humans, who are their common cohabitants. Children (smaller in size) vocalize with higher F0 than adult women who are themselves higher pitched (and typically smaller in size) than adult men [32], meaning that dogs could benefit from being attuned to this difference, via an internalized pitch-size correspondence rule.

Our findings of a pitch-size correspondence in dogs show this is not unique to humans or non-human primates, in turn suggesting it may be based on a shared common mechanism. Cross-sensory correspondences such as those shown here could, in theory, be explained by Bayesian ‘coupling priors’ where perception is affected by expectations derived from the environment [33]. For example, smaller animals tend to emit higher pitch vocalizations, and smaller inanimate objects emit a higher pitch sound when struck (c.f. small versus large bells). As well as this statistical account—based on the regularities of sound-size co-occurrence in the environment [10]—the pitch-size correspondence may also be due to the organization of the nervous system (see [34] for the first similar suggestion in humans). We propose that future studies might examine these pairings in dogs of different ages. If dogs show weaker evidence of the pitch-size sensitivity as young puppies, we might infer that this stems from their limited exposure to world statistics and will give more weight to the statistical theory of these correspondences.

In our study, dogs reliably chose the correct objects on congruent trials when there was no conflict between the size of the object making the sound and the pitch of the sound. However, their performance in incongruent trials was not different to random choice. This shows sensitivity to crossmodal correspondences, perhaps because incongruent trials provide confusing signals to the dog. While their prior training guides them towards the sound, their crossmodal intuitions provide conflicting internal information, pointing the dog away from the sound-source, towards the object ‘matching’ in pitch. Together these conflicts may leave the dog unable to detect the correct object at levels different to chance in incongruent trials. This result is similar to a finding in humans by [35]: human participants struggled to correctly locate a sound if it was not coming from where an object of a congruent size was positioned—instead attributing the source of the sound to the congruent object. Hence, the pitch-size correspondence might facilitate the spatial integration of congruent multisensory stimuli by perceptually binding them, which could explain why on some incongruent trials the dogs in our study chose the congruent but incorrect object. Alternatively, the conflict between object and mismatching sound may have disrupted the dogs' processing of the spatial location of the sound, leaving them to choose randomly.

We also found that dogs showed longer latencies to move on incongruent trials as opposed to congruent trials. Hence although congruency did not affect how quickly the dogs ultimately arrived at the object, it did affect how quickly they made their first move (i.e. slower on incongruent trials). This mirrors findings from human experiments where participants have slower reaction times on trials where the irrelevant auditory stimulus does not intuitively match the size of the visual stimulus that they were classifying as big or small [30,31]. However, while the human studies suggest that the congruency in the pitch-size pairings has a facilitatory effect on performance [30], our findings leave us uncertain as to whether congruency facilitates responses, or incongruency inhibits them—or indeed both. Future studies should include a baseline score (where e.g. the dog is presented with two objects of the same size and a sound matched in pitch to the size of the objects) to ascertain whether congruency facilitates dogs’ responses or indeed lack of congruency hampers.

To the best of our knowledge, this is the first evidence of abstract pitch-size correspondences in a non-human animal. If these correspondences are the result of world statistics, where the correspondence is learnt from observing common occurrences of it, their existence in this non-human species is perhaps not surprising, given that dogs share the same environment with humans and are exposed to many of the same regularities in that environment. Our future work will aim to establish whether dogs show correspondences which *cannot* so easily be explained by world statistics—e.g. pitch-luminance (where humans preferentially match light colours with high pitches and dark colours with low pitches). If dogs are sensitive to these correspondences, this might suggest a further shared mammalian characteristic, and potential pre-natal organization and development of the nervous system for multimodal perception and integration.

In conclusion, our study shows that dogs form the same pitch-size crossmodal correspondence as humans. While we cannot yet identify the routes via which this correspondence arises, we suggest that further work investigating non-human animals could help to reveal the commonalities in how the perceptual systems of humans and non-humans respond to crossmodal stimuli.

## Footnotes

1 It might be argued that the opposition of high and low pitch is not apparent to the dog on the first trial. However, sounds also stand in opposition to the training sound. Hence, even on the first trial, our ‘high’ pitch sound is higher than the training sound.

## Data accessibility

The data is available on Dryad and is accessible under the following link: https://doi.org/10.5061/dryad.6q573n60g [36]. The data are provided in the electronic supplementary material [37].
